# Q&A with Editorial Board Member Professor Wei Zhang

**DOI:** 10.1038/s42004-023-00916-3

**Published:** 2023-06-12

## Abstract

Professor Wei Zhang answers questions on his scientific career, scientific developments he is excited about, directions electrochemical energy storage and conversion technologies should focus on, as well as his experience of being an Editorial Board Member for *Communications Chemistry*.

Wei Zhang is currently a full Professor (Tang Auchin Scholar-Leading Professor since 2020) and the Director of the Electron Microscopy Center at Jilin University, China. He is Chairman of the Jilin Electron Microscopy Society and a Fellow of the Royal Society of Chemistry.

**Figure:**
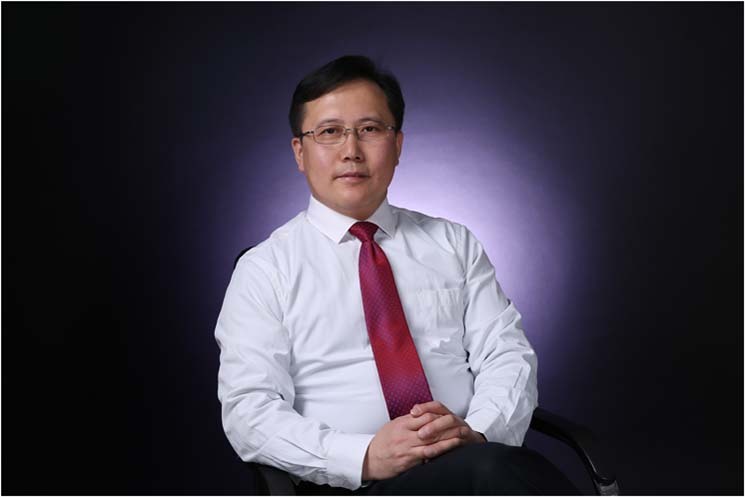
Wei Zhang

Wei earned his PhD from the Institute of Metal Research, Chinese Academy of Sciences, in 2004. He held academic positions at the National Institute for Materials Science (Japan), the Samsung Advanced Institute of Technology (South Korea), the Fritz Haber Institute of the Max Planck Society (Germany), the Technical University of Denmark, and the CIC Energigune (Spain). In 2016, Wei was acknowledged as Ikerbasque Research Professor in Spain. He elucidated the surface termination of the industrially important complex oxide catalyst MoVTeNbO_x_ via real-space observation^1^. Wei invented a nanopatterning method for direct writing on graphene “paper” by manipulating electrons as “invisible ink”^2^. In an influential publication in *Nature Communications* in 2017, Wei and team reported a battery-mimicking mechanism in supercapacitors^3^. He proposed a thin-film theory-based strategy of suppressing lithium dendrites via tuning surface energy^4^, and a diffusionless-transformation-like conversion reaction achieving bulk-utilization^5^ of electrode materials for pseudocapacitance. His current research focuses on surface and interface architecture chemistry of advanced materials toward applications in electrochemical energy storage/conversion and heterogeneous catalysis.

Why did you choose to be a scientist?

Being a scientist is a noble profession that involves exploring the mysteries of our world. Through science, one can better understand the world and the universe. From a young age, I have been interested in the wonders of science and have nurtured a dream to become a scientist. My goal is to constantly improve myself as a scientist and unravel more of the mysteries of our world.

What scientific development are you currently most excited about?

Advanced electron microscopy and spectroscopy techniques have been a focal point of my research since I began pursuing my M.D. and Ph.D. at the Institute of Metal Research Chinese Academy of Sciences in 1997, specifically in the microanalysis of materials. What I am most excited about is the ability to utilize the “eyes” of electron microscopy to observe and identify well-known findings, e.g., quasicrystals as first reported by Daniel Shechtman^6^, carbon nanotubes discovered by Sumio Iijima^7^ and more. My present objective is to explore the relationship between sub-Ångström-, nano-, and micro-scale architected structures and the performance of such functional materials, as well as their application in various scenarios such as large-scale electrochemical energy conversion and storage devices.

What direction do you think your research field should go in?

Cutting-edge electrochemical energy storage and conversion technologies, such as batteries, supercapacitors, as well as electrocatalysis, offer exceptional efficiency in energy utilization and may also satisfy practical demands: these technologies provide basic raw materials and fuels for chemical industries that have a crucial effect on the economy and people’s well-being. In light of this, the goal of my research goes towards promoting a sustainable societal development.

What attracted you to becoming an Editorial Board Member for *Communications Chemistry*?

As a scientist, upholding a mindset consistently focused on curiosity, skepticism, evaluation, communication, and verification is crucial. This necessitates collaboration with other professional scientists and the community. *Communications Chemistry* publishes cutting-edge research from all fields of chemistry, and this is what initially enticed me to become an Editorial Board Member. Collaborating with other scientists in the generation of innovative ideas is a reliable way of challenging myself. This mindset creates a mutually beneficial route for the advancement of the international chemistry community.

What have you gotten out of the experience of being an Editorial Board Member for *Communications Chemistry*?

As an Editorial Board Member, I have had the opportunity to interact and engage with scientists from diverse fields of chemistry. High-level work needs thorough preparation, comprehensive analysis, and experimentation or theory — often both. Such work bears the weighty responsibility of advancing global development of chemical technologies. I have been able to integrate and apply ideas from various disciplines to my own area of research, giving me fresh inspiration. This has also given me cause to re-examine the challenges I have encountered in my research, opportunities to discover corresponding solutions, and effective guidance.

How do your editorial responsibilities integrate with your academic role?

My editorial responsibilities and academic roles are complementary, assisting me in approaching scientific issues with a dialectical and global perspective and fueling my own academic innovation. Such a great responsibility also motivates me to focus on interdisciplinary research and to share my expertize with those in other fields. Handling submissions gives me a sense of accomplishment and helps me stay up to date with the latest global developments in chemistry.

What do you see as the role of *Communications Chemistry* in the scientific community?

Science knows no borders, and disciplines know no boundaries. *Communications Chemistry* stands as a prime example of this, publishing some of the most influential research across various fields of chemistry that inspire and influence beyond their respective domains. As long as one possesses great ideas and makes significant research contributions, this platform provides ample opportunities to share one’s expertize, thereby inspiring everyone. Simultaneously, *Communications Chemistry* ensures that authors reach a broad audience by publishing through its prestigious Nature Portfolio brand and implementing its Open Access policy. *Communications Chemistry* provides an outlet for valuable interdisciplinary chemistry research topics that are essential for researchers to understand the direction of crucial fields and for decision-makers to address key issues.

*This interview was conducted by the editors of Communications Chemistry*.

## References

[1] Zhang W, Trunschke A, Schlögl R, Su D (2010). Real-space observation of surface termination of a complex metal oxide catalyst. Angew. Chem. Int. Ed..

[2] Zhang W (2013). Direct writing on graphene ‘paper’ by manipulating electrons as ‘invisible ink’.. Nanotechnology.

[3] Deng T (2017). Atomic-level energy storage mechanism of cobalt hydroxide electrode for pseudocapacitors. Nat. Commun..

[4] Wang D (2017). Towards high-safe lithium metal anodes: suppressing lithium dendrites via tuning surface energy. Adv. Sci..

[5] Dong T (2023). Diffusionless-like transformation unlocks pseudocapacitance with bulk utilization: reinventing Fe_2_O_3_ in alkaline electrolyte. Energy Environ. Mater..

[6] Shechtman D, Blech I, Gratias D, Cahn JW (1984). Metallic phase with long-range orientational order and no translational symmetry. Phys. Rev. Lett..

[7] Iijima S (1991). Helical microtubules of graphitic carbon. Nature.

